# Supplementary material for: Heavy Metals in Soil around a Typical Antimony Mine Area of China: Pollution Characteristics, Land Cover Influence and Source Identification
Source: Int J Environ Res Public Health. 2023 Jan 25;20(3):2177. doi: 10.3390/ijerph20032177 (PMC9915987; doi:10.3390/ijerph20032177)
Supplement: Supplementary file 1 [file ijerph-20-02177-s001.zip › ijerph-2144386-supplementary.pdf]

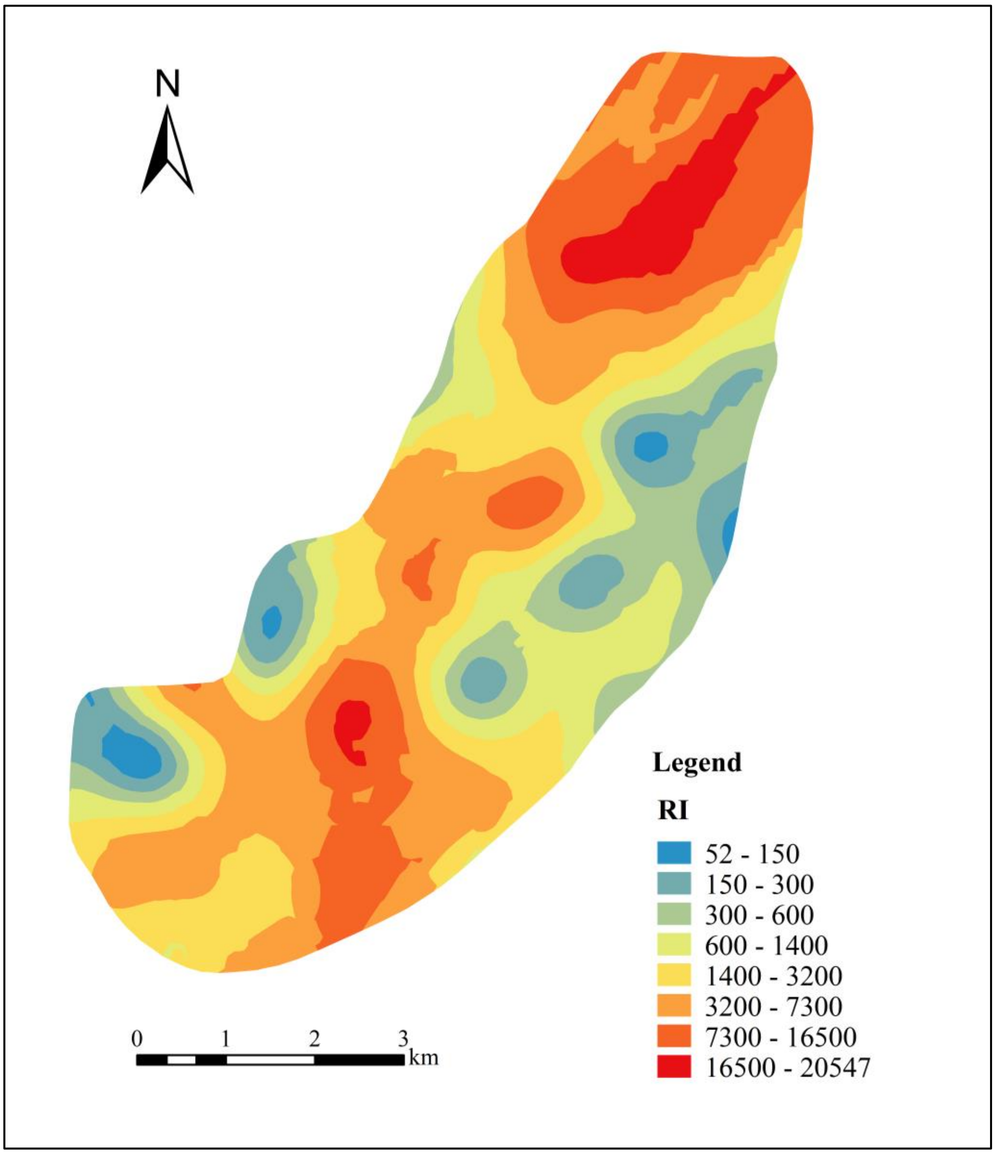

Figure S1. Distribution map of the potential ecological risk index (*RI*) of soil heavy metal contamination of the study area.

Table S1. Ordinary Kriging interpolation prediction errors of soil heavy metal concentrations in the study area.

Element	Mean error	Mean standardized error	Average standard error	Root mean square error	Root mean square standardized error
Sb	−0.120	−0.008	1043.680	1101.115	1.050
As	−0.230	−0.005	47.370	51.340	1.082
Cu	0.310	0.010	25.260	21.873	0.904
Zn	−0.450	−0.006	219.40	197.64	0.964
Pb	0.800	0.010	44.890	47.174	1.093
Cr	−0.490	−0.016	32.873	35.110	1.050
Ni	−0.480	−0.016	19.480	24.187	1.220
Cd	−0.040	−0.002	5.501	8.095	1.380
Hg	0.085	0.016	5.442	5.054	0.954

The judging criterion: the absolute value of the mean error and the mean standardized error were close to 0, the average standard error is close to root mean square error, and the root mean square standardized error is close to 1.

Table S2. Classification criteria for Nemerow's comprehensive pollution index (P_s).

P_s value	Class	Contamination situation
$P_s \leq 0.7$	1	Safety
$0.7 < P_s \leq 1.0$	2	Precaution
$1.0 < P_s \leq 2.0$	3	Slightly polluted
$2.0 < P_s \leq 3.0$	4	Moderately polluted
$P_s > 3.0$	5	Severely polluted

Table S3. Classification criteria of the potential ecological risk for heavy metal contamination.

E_r^i value	Class	Level of single heavy metal ecological risk	RI value	Class	Level of comprehensive potential ecological risk
$E_r^i < 40$	1	Low risk	$RI < 150$	1	Low risk
$40 \leq E_r^i < 80$	2	Moderate risk	$150 \leq RI < 300$	2	Moderate risk
$80 \leq E_r^i < 160$	3	Considerable risk	$300 \leq RI < 600$	3	Considerable risk
$160 \leq E_r^i < 320$	4	High risk	$RI \geq 600$	4	Very high risk
$E_r^i \geq 320$	5	Very high risk			

Table S4. Correlation analysis of soil heavy metals of the study area.

	Sb	As	Hg	Cr	Cd	Pb	Cu	Zn	Ni
Sb	1								
As	0.256	1							
Hg	0.465	0.403 **	1						
Cr	−0.171	0.065	−0.034	1					
Cd	0.059	0.115	0.000	−0.017	1				
Pb	0.269	0.505 **	0.822 **	0.108	0.133	1			
Cu	0.069	0.250	0.051	0.630 **	0.174	0.384 *	1		
Zn	0.293	0.391 *	0.067	0.130	0.470 **	0.329 *	0.422 **	1	
Ni	−0.223	−0.135	−0.198	0.768 **	0.110	−0.064	0.355 *	0.127	1

**: At 0.01 level (double tail), the correlation is significant. *: At 0.05 level (double tail), the correlation is significant.
